# SARS-CoV-2 Variants BQ.1 and XBB.1.5 in Wastewater of Aircraft Flying from China to Denmark, 2023

**DOI:** 10.3201/eid2912.230717

**Published:** 2023-12

**Authors:** Amanda Gammelby Qvesel, Marc Bennedbæk, Nicolai Balle Larsen, Vithiagaran Gunalan, Lene Wulff Krogsgaard, Morten Rasmussen, Lasse Dam Rasmussen

**Affiliations:** Author affiliation: Statens Serum Institut, Copenhagen, Denmark

**Keywords:** COVID-19, respiratory infections, severe acute respiratory syndrome coronavirus 2, SARS-CoV-2, SARS, coronavirus disease, coronavirus, wastewater, sequence analysis, aircraft, COVID-19 nucleic acid testing, PCR, whole-genome sequencing, viruses, zoonoses, China, Denmark

## Abstract

We analyzed wastewater samples from 14 aircraft arriving in Denmark directly from China during January 9–February 12, 2023. Wastewater from 11 aircraft was SARS-CoV-2–positive by PCR; 6 predominantly contained BQ.1 and XBB.1 subvariants. Wastewater-based surveillance can contribute to public health monitoring of SARS-CoV-2 and other emerging infectious agents.

Relaxation of China’s zero-COVID policy in December 2022 led the European Centre for Disease Prevention and Control to recommend several nonpharmaceutical interventions to curb COVID-19 spread and monitor any emerging SARS-CoV-2 variants; those interventions included wastewater-based surveillance (*1*). We report results of subsequent wastewater surveillance of aircraft arriving at Copenhagen Airport in Copenhagen, Denmark, directly from Beijing or Shanghai, China.

During weeks 2–6 of 2023 (January 9–February 12), a total of 14 aircraft arrived at Copenhagen Airport from China. A service truck extracted waste from the aircraft by using vacuum pressure, after which a rinsing program was performed, and the disinfectant Idu-Flight (Brenntag Nordic A/S, https://www.brenntag.com) was added to the waste tank. Wastewater samples were collected as grab samples from the service truck and immediately transported to Statens Serum Institut in Copenhagen for analysis.

The pH value of the sample material ranged from 9–10 because of the addition of Idu-Flight. Idu-Flight contains the active ingredients glutaraldehyde and benzalkonium chloride; the disinfectant is expected to negatively affect the stability of virus particles and hinder amplification of RNA sequences. We adjusted the samples to pH 7.5–8.5 by using HCl and homogenized them by vigorous vortexing. We split the 14 samples into a total of 43 aliquots and then centrifuged those at either 4,000 × *g* or 10,000 × *g* for 10 min to pellet solid material. For the first aliquot from aircraft AC1, we analyzed 10 mL of sample material without any centrifugation; for all other samples, we analyzed 10 mL of supernatant after centrifugation. We purified viruses by using NanoTrap Microbiome A particles (Ceres Nanosciences Inc., https://www.ceresnano.com) and RNA by using Maxwell RSC Cartridges (Promega Corporation, https://www.promega.com). We performed quantitative reverse transcription PCR (qRT-PCR) in technical triplicate by using the GoTaq Enviro kit (Promega) and the US Centers for Disease Control and Prevention N2 primer/probe for SARS-CoV-2 detection (Table; Appendix Table).

**Table:** Sequencing results for SARS-CoV-2–positive wastewater samples in study of SARS-CoV-2 variants BQ.1 and XBB.1.5 in wastewater of aircraft flying from China to Denmark, 2023*

Aircraft ID	Arrival week	Raw sequence reads	Mapped sequence reads	Genome (spike) coverage,† %	Pangolin lineage‡ (% abundance)
AC1	2	16,699,590	948,407	85.5 (49.5)	XBB.1.1 (21), BA.5 (9), XBB.2 (7), XBB.1 (7)
AC2	2	15,892,650	122,882	48.5 (17.6)	NA
AC3	3	14,467,192	725,320	93.1 (90.7)	BQ.1 (60)
AC4	4	13,463,062	5,584	10.3 (8.1)	NA
AC5	4	25,193,066	607,011	83.9 (88.7)	XBB.1.5 (96)
AC6	4	5,215,440	135	16.7 (0.0)	NA
AC7	5	15,691,594	960,609	82.9 (91.4)	XBB.1.5 (99)
AC8	5	2,749,174	102	0.0 (0.0)	NA
AC9	6	5,880,410	14,457	14.0 (9.1)	NA
AC10	6	13,244,030	1,306,993	98.1 (95.2)	XBB.1.5 (59), XBB.2 (18), XBB.1 (18)
AC11	6	1,132,399	1,127,995	98.1 (95.2)	XBB.1.5 (97)
EC§	NA	4,050,388	370	0.5 (0.0)	NA

*Wastewater was collected from aircraft during January 9 (week 2)–February 12 (week 6), 2023. Samples from each aircraft were combined and reverse transcription quantitative PCR and sequencing were performed; only 1 sample each was collected from AC6, AC8, and AC9. Full table of results for each aliquot is available in the Appendix). EC, extraction control; ID, identification; NA, not applicable. †Coverage percentages are provided for the full genome sequence and for the spike protein gene. ‡Lineages according to Pangolin software (https://cov-lineages.org). §Water sample was extracted and sequenced as a control.

Of the 43 qRT-PCR reactions, 31 (72%) were positive for SARS-CoV-2, representing 11 aircraft. We conducted whole-genome sequencing of samples from those 11 aircraft by using the Illumina MiSeq platform (https://www.illumina.com) according to the ARTIC protocol; we generated 2 × 150-bp paired-end reads by using the ARTIC 4.1 primer scheme (*2*). Wastewater raw reads are available from the European Nucleotide Archive (https://www.ebi.ac.uk/ena; accession no. PRJEB66221). We trimmed reads by using Trim Galore with default settings (*3*; https://zenodo.org/record/5127899). We removed human sequence reads by using the BWA-MEM alignment algorithm with default settings (H. Li, unpub. data, http://arxiv.org/abs/1303.3997) and the human genome reference build GRCh38. We then used BWA-MEM with default settings to map SARS-CoV-2 reads to the SARS-CoV-2 wild-type reference genome (GenBank accession no. MN908947.3). We performed primer trimming by using iVar with a minimum read length of 30 nt (*4*) and estimated SARS-CoV-2 lineage abundance in each sample by using Freyja; depth cutoff was 10×, and the lineage abundance filter was 5% (*5*). We used a 50% coverage minimum across the genome as the threshold for lineage calling. We obtained sequencing results for 13 (42%) of 31 SARS-CoV-2–positive samples (Appendix Table). 

We analyzed sequence reads for each sample aliquot and also after combining raw reads for each aircraft (Table; Appendix Table). When reads were combined for each aircraft, we found that the SARS-CoV-2 BQ.1 variant was dominant in wastewater of 1 aircraft, and XBB.1 variants were dominant in wastewater of 5 aircraft; the XBB.1.5 subvariant was dominant in 4 of those 5 aircraft (Table). The discovery of predominant XBB subvariants (dominant in Europe and the United States during the study period) in the aircraft samples contrasts with variant data uploaded to the GISAID database (https://www.gisaid.org) from China within the same time frame, which were mainly subvariants BA.5.2.48 and BF.7.14 (*6*).

For wastewater-based surveillance, limited information is generally available regarding the persons who contributed to the samples, and, consequently, data related to travel history and place of residence are lacking. Because of the lack of supporting information for passengers, the SARS-CoV-2 variants observed in wastewater-based surveillance of aircraft arriving in Copenhagen might have come from passengers infected outside of China.

In conclusion, our findings indicate that the largely infection-naive population of China might not have comprised a strong selective force driving SARS-CoV-2 toward variants with immune evasive features, such as BQ.1 and XBB. Thus, if BQ.1 and XBB.1.5 subvariants were indeed circulating in China to the extent suggested by our analysis, their dominance in wastewater samples might have occurred because of a founder effect in selected communities instead of those variants arising in China. Since January and February 2023, XBB has become the dominant variant in sequence data from China (*6*). No new variants have been identified, but our study highlights the potential for wastewater-based surveillance to monitor virus spread among airline passengers in a cost-effective, anonymous, and noninvasive manner and to potentially identify circulating variants. This method can be rapidly modified to include other emerging infectious agents and can contribute substantially to future public health surveillance.

Appendix Additional information for SARS-CoV-2 variants BQ.1 and XBB.1.5 in wastewater of aircraft flying from China to Denmark, 2023.
