# Supplementary material for: SARS-CoV-2 Variants BQ.1 and XBB.1.5 in Wastewater of Aircraft Flying from China to Denmark, 2023
Source: Emerg Infect Dis. 2023 Dec;29(12):2559–61. doi: 10.3201/eid2912.230717 (PMC10683830; doi:10.3201/eid2912.230717)
Supplement: Appendix — Additional information for SARS-CoV-2 variants BQ.1 and XBB.1.5 in wastewater of aircraft flying from China to Denmark, 2023. [file 23-0717-Techapp-s1.pdf]

Article DOI: https://doi.org/10.3201/eid2912.230717

EID cannot ensure accessibility for supplementary materials supplied by authors. Readers who have difficulty accessing supplementary content should contact the authors for assistance.

SARS-CoV-2 Variants BQ.1 and XBB.1.5 in Wastewater of Aircraft Flying from China to Denmark, 2023

Appendix

Appendix Table. Extended sequencing results for individual SARS-CoV-2–positive wastewater samples in study of SARS-CoV-2 variants BQ.1 and XBB.1.5 in wastewater of aircraft flying from China to Denmark, 2023*

Aircraft ID*	Arrival week	Ct	Raw sequence reads	Mapped sequence reads	Genome (spike) coverage,† %	Pangolin lineage‡ (% abundance)
AC1	2	37.2	4,424,432	199,962	62.2 (20.5)	BA.2.85 (58), XBB.1.7 (27)
		36.8	4,446,070	340,210	54.3 (38.8)	BA.2.10.1 (32), BA.2.10 (32)
		38.3	2,760,420	23,213	5.1 (10.5)	NA
		35.4	5,068,668	385,022	47.7 (20.8)	NA
Combined AC1 samples	2	NA	16,699,590	948,407	85.5 (49.5)	XBB.1.1 (21), BA.5 (9), XBB.2 (7), XBB.1 (7)
AC2	2	35.4	4,602,774	107,184	42.1 (17.6)	NA
		36.8	4,167,118	11,265	6.0 (0.0)	NA
		37.8	3,629,122	2,983	2.4 (0.0)	NA
		37.6	3,493,636	1,450	1.2 (0.0)	NA
Combined AC2 samples	2	NA	15,892,650	122,882	48.5 (17.6)	NA
AC3	3	34.9	1,486,100	125,201	87.6 (51.1)	BQ.1 (37), BQ.1.17 (8), BQ.1.1.28 (6)
		34.2	4,561,054	240,781	63.1 (67.3)	BQ.1.24 (15), BQ.1.19 (15), BQ.1.11 (15), BQ.1.15 (15), BQ.1.20 (15), BQ.1 (15)
		35.4	3,868,320	139,310	77.4 (75.6)	BQ.1.17 (15), BQ.1.26 (15), BQ.1.19 (15), BQ.1.11 (15), BQ.1 (15), BQ.1.20 (15)
		34.3	4,551,718	220,028	63.5 (8.1)	BE.1.1 (32), BQ.1 (14), BQ.1.26 (14), BQ.1.11 (14), BQ.1.15 (14)
Combined AC3 samples	3	NA	14,467,192	725,320	93.1 (90.7)	BQ.1 (60)
AC4	3	38.4	3,655,710	662	1.2 (8.1)	NA
		38.4	3,194,498	225	1.1 (0.0)	NA
		38.4	3,676,300	3,474	2.2 (0.0)	NA
		38.3	2,936,554	1,223	2.4 (0.0)	NA
Combined AC4 samples	3	NA	13,463,062	5,584	10.3 (8.1)	NA
AC5	4	38.9	6,993,416	105,986	52.9 (43.7)	XBB.1.5 (99)
		37.8	5,253,686	204,214	32.8 (41.5)	NA
		37.2	5,716,928	66,675	17.0 (18.0)	NA
		37.9	7,229,036	230,136	51.2 (79.8)	XBB.1.5 (96)
Combined AC5 samples	4	NA	25,193,066	607,011	83.9 (88.7)	XBB.1.5 (96)
AC6	4	38.4	5,215,440	135	16.7 (0.0)	NA
AC7	5	38.7	3,306,180	3,2013	39.8 (31.8)	NA
		38.4	4,204,666	124,643	51.2 (34.5)	XBB.1.5 (99)
		37.9	4,719,822	601,107	56.8 (49.4)	XBB.1.5 (97)
		38.3	3,460,926	202,846	26.4 (55.6)	NA
Combined AC7 samples	5	NA	15,691,594	960,609	82.9 (91.4)	XBB.1.5 (99)
AC8	5	38.3	2,749,174	102	0.0 (0.0)	NA
AC9	6	38.0	5,880,410	14,457	14.0 (9.1)	NA
AC10	6	34.4	5,970,934	102,160	68.5 (57.7)	XBB.1.5 (97)

Aircraft ID*	Arrival week	Ct	Raw sequence reads	Mapped sequence reads	Genome (spike) coverage,† %	Pangolin lineage‡ (% abundance)
		35.4	7,273,096	1,204,834	97.2 (95.2)	XBB.1.5 (59), XBB.1 (18), XBB.2 (18)
Combined AC10 samples	6	NA	13,244,030	1,306,993	98.1 (95.2)	XBB.1.5 (59), XBB.2 (18), XBB.1 (18)
AC11	6	36.1	5,092,066	222,781	93.4 (82.4)	XBB.1.5 (96)
		35.1	6,231,930	905,214	41.7 (95.2)	NA
Combined AC11 samples	6	NA	1,132,399	1,127,995	98.1 (95.2)	XBB.1.5 (97)
EC§	NA	NA	4,050,388	370	0.5 (0.0)	NA

*Wastewater was collected from aircraft during January 9 (week 2)–February 12 (week 6), 2023. For combined samples, all raw reads for that aircraft were pooled before analysis. Only 1 sample each was collected from AC6, AC8, and AC9. Ct, cycle threshold; EC, extraction control; ID, identification; NA, not applicable.
†Coverage percentages are provided for the full genome sequence and for the spike protein gene.
‡Lineages according to Pangolin software (https://cov-lineages.org).
§Water sample was extracted and sequenced as a control.
